# Artificial Bone Graft Substitutes for Curettage of Benign and Low-Grade Malignant Bone Tumors: Clinical and Radiological Experience with Cerasorb

**DOI:** 10.1007/s43465-023-00919-1

**Published:** 2023-06-22

**Authors:** Ulrike Susanne Wittig, Jörg Friesenbichler, Bernadette Liegl-Atzwanger, Jasminka Igrec, Dimosthenis Andreou, Andreas Leithner, Susanne Scheipl

**Affiliations:** 1 Department of Orthopaedics and Trauma, Medical University of Graz, Auenbruggerplatz 5, Graz, Austria; 2 Department of Trauma Surgery, Wiener Neustadt State Hospital, Wiener Neustadt, Austria; 3 Diagnostic and Research Center for Molecular Biomedicine, Diagnostic and Research Center for Pathology, Medical University of Graz, Graz, Austria; 4 Division of General Radiology, Department of Radiology, Medical University of Graz, Graz, Austria

**Keywords:** Artificial bone graft substitutes, ABGS, Bone tumors, Curettage

## Abstract

**Background:**

Artificial bone graft substitutes (ABGS) for curettage of bone tumors are becoming increasingly popular. The aim of this retrospective analysis was to determine the efficacy of the ABGS Cerasorb (Curasan-AG, Kleinostheim, Germany), a beta-tricalcium phosphate (beta-TCP), concerning resorption profile, bone healing, and remodeling after surgery and to evaluate potential complications.

**Methods:**

Forty-three patients suffering from benign and low-grade malignant bone tumors were treated with curettage and refilling of the bony cavity using the ABGS Cerasorb between 2018 and 2021 and included in the final analysis. Clinical follow-up exams with X-rays in two planes were performed 6 weeks, 3 months, 6 months, and 1 year after surgery.

**Results:**

After a mean follow-up period of 14.6 months, radiological consolidation following curettage was observed in all patients. Total resorption was observed in 16.3% of patients; in the other 83.7%, resorption was partial. In four patients, of whom two had a tumor in the distal femur and two in the humeral diaphysis, fractures occurred within 6 weeks after primary surgery.

**Conclusion:**

In conclusion, the beta-TCP Cerasorb seems to be a reliable bone graft substitute with low complication rates and is a suitable alternative to autologous bone grafts or allografts. Nonetheless, it shows a tendency of delayed resorption.

**Level of Evidence:**

III; retrospective cohort study.

## Introduction

Benign and low-grade malignant bone tumors are usually treated with intralesional curettage. Subsequently, the bony cavity can be left empty or it can be filled with autologous or allogenic materials. The aim of filling with grafts is full bony ingrowth and remodeling into mature bone [1, 2]. Surgical adjuvants such as artificial bone graft substitutes (ABGS) have become increasingly popular, as they do not involve donor site morbidity. Known side effects of harvesting autologous bone grafts from the iliac crest include risk of infection, delayed wound healing, neuro-vascular injuries, heterotopic ossification, and local pain [2, 3]. On the other hand, allogenic materials have been reported to be immunogenic and might transfer infectious diseases, with complication rates for infection of up to 12.2% [4, 5].

Several ABGS are available, including demineralized bone matrix, bone graft extenders, and bone morphogenic proteins. Demineralized bone matrix contains proteins gained from processed cadaver bone without minerals. Bone morphogenic proteins induce new bone formation by stimulating osteoblastic differentiation. Bone graft extenders comprise ceramics (e.g., hydroxyapatite), salts (e.g., calcium sulphate, tricalcium phosphate), and synthetic products such as polymethylmethacryclate (PMMA). They are shapeable and, therefore, can be used to fill irregularly shaped defects. Moreover, they are accessible unrestrictedly and do not involve the risk of transmitting infectious diseases [2, 6–10]. Tricalcium phosphates (TCP) are characterized by their biocompatibility, favorable resorption properties, and osteoconductivity [11, 12].

Cerasorb (Curasan-AG, Kleinostheim, Germany) is a pure-phase beta-tricalcium phosphate (β-TCP) with precisely defined physical and chemo-crystalline properties and a high level of uniformity of chemical composition and purity. In contrast to alpha-tricalcium phosphates (α-TCP), β-TCP is thermodynamically stable in a biological environment and within a normal temperature range. Biodegradation is faster than that of α-TCP. The safety profile of Cerasorb has been checked in various preclinical and clinical studies [13–15].

The aim of this retrospective analysis was to determine the efficacy of the artificial bone graft substitute Cerasorb concerning resorption profile, bone healing, and remodeling after surgery and to evaluate potential complications.

## Materials and Methods

At the initial phase of patient recruitment, 55 potential patients with benign and low-grade malignant bone tumors treated at a single center between November 2018 and July 2021 with curettage and filling with Cerasorb were found. However, 12 patients were lost for follow-up within the first 4 months after surgery, eventually leaving 43 patients for analysis, who were included in this study and retrospectively reviewed. Prior to surgery, written informed consent was obtained from all patients, who were informed about advantages and disadvantages of filling defects after curettage with artificial bone graft substitutes. The study was approved by the ethics committee of the Medical University of Graz (EK 34-139 ex 21/22) prior to conduction of the study. Only patients with benign and low-grade malignancies were included. Conventional radiographs as well as CT and MRI scans of the lesions were examined by an experienced tumor radiologist to classify the tumors. If classification and dignity of the lesions were unclear, biopsies were performed and looked at by expert bone tumor pathologists. Surgery was chosen over a conservative treatment approach due to lesion's size, the potential of tumor progression, and risk of pathological fracture. Clinical follow-up exams with X-rays in two planes were performed 6 weeks, 3 months, 6 months, and 12 months after surgery. All surgeries were carried out by experienced orthopedic tumor surgeons.

Following surgical exposure of the affected bone, an osseous window was created to gain access to the lesion. Curettage was performed under fluoroscopic visualization using a c-arm. The curetted tissue was sent for histopathological examination. All diagnostic evaluations were performed by expert bone tumor pathologists and classified by the WHO Classification for Bone and Soft Tissue Tumors 2020 [16]. The ABGS was prepared according to the respective manufacturer's instructions, injected into the cavity and allowed to dry for several minutes. Subsequently, the osseous window was re-attached. In 13 patients, a protective plate osteosynthesis was performed to enhance stability. Finally, the layers of the wound were closed.

Parameters assessed in this study included time required for osseous consolidation and resorption, local recurrence rates, and complications. Surgical complications were reported according to the classification system by Goslings and Gouma as follows: 0 (no harm), 1 (temporary disadvantage, no reoperation), 2 (recovery after reoperation), 3 (permanent damage/disability), 4 (death), and 5 (unclear as a result of untimely death) [17]. Assessment of osseous integration, resorption and bony healing was performed according to the modified Neer score [18, 19].

## Results

**Fig. 1:**
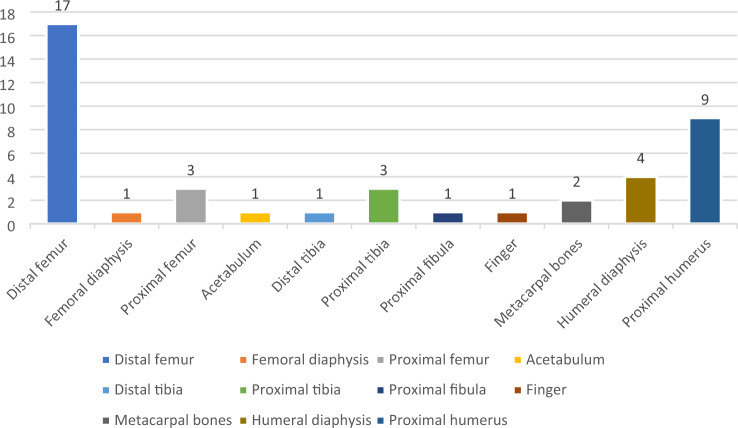
A graphical comparison of bone tumor localizations

**Fig. 2:**
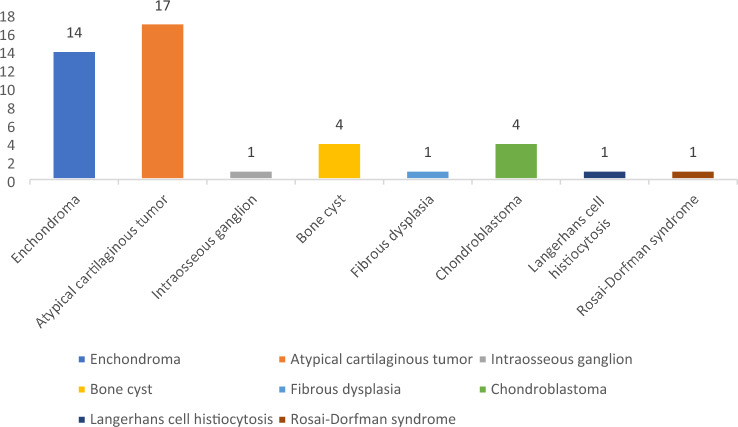
A graphical comparison of tumor entities analyzed in the present study

Altogether, 43 patients who had received curettage of benign or low-grade malignant bone tumors and filling with the ABGS Cerasorb were included in this study. The study cohort consisted of 21 male and 22 female patients. Patients’ mean age at surgery was 42 years (range 15–70 years). Seventeen lesions were located in the distal femur, one was in the femoral diaphysis, one was in the proximal fibula, three lesions were in the proximal tibia, one in the distal tibia, one was in the acetabulum, two in the metacarpal bones, and one in the medial phalanx of the fifth finger, three lesions were in the proximal femur, four were located in the humeral diaphysis, and nine lesions were located in the proximal humerus. A summary of tumor locations is depicted in Fig. 1. The tumor entities comprised enchondromas, simple or juvenile bone cysts, atypical cartilaginous tumors, chondroblastomas, one case of Langerhans cell histiocytosis, one intraosseous ganglion, fibrous dysplasia and a case of Rosai–Dorfman syndrome, the frequency of which is presented graphically in Fig. 2. The mean filling volume applied to the bony cavities was 40.2 ml (range 5–100 ml). The mean maximum diameter of the lesions was 5.6 cm (range 1.8–14.5).

According to the bone healing classification system by Neer et al. [18, 19], all lesions were grade I lesions, indicating that curetted tumors require no further treatment. The study collective included no grade II, III or IV lesions. However, in nine cases, an additional plate osteosynthesis was performed to achieve more stable conditions. In two patients, an additional structural allograft was added. This was decided intraoperatively by the performing surgeon.

**Fig. 3:**
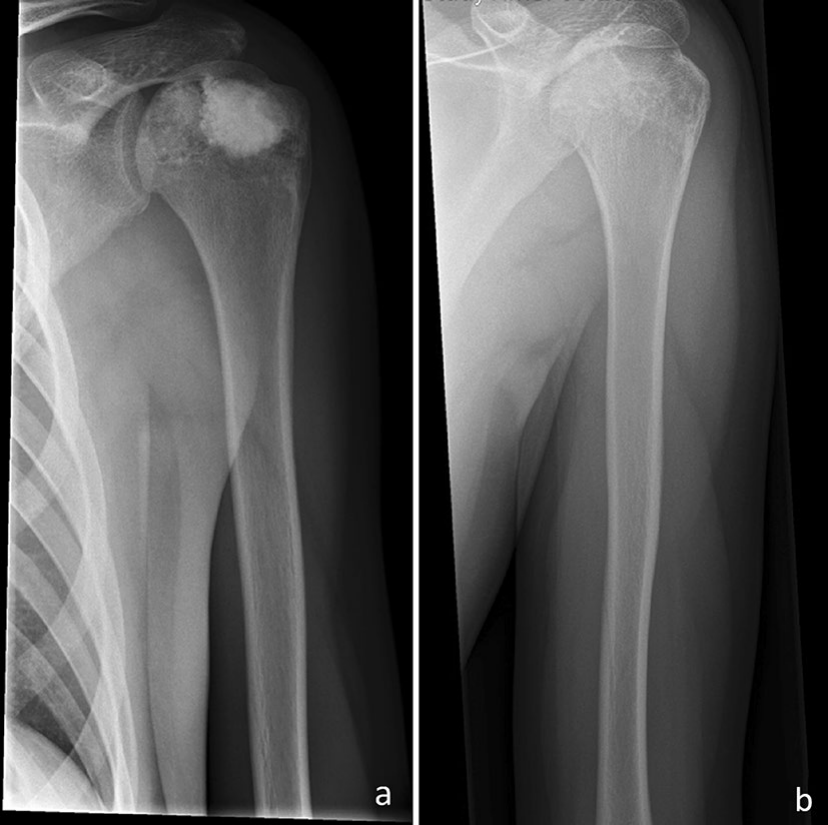
**a** X-ray of a 15-year-old male patient a few days after curettage of a chondroblastoma of the left proximal humerus. **b** Follow-up radiographs 12 months following index surgery showing complete resorption of the artificial bone graft substitute

**Fig. 4:**
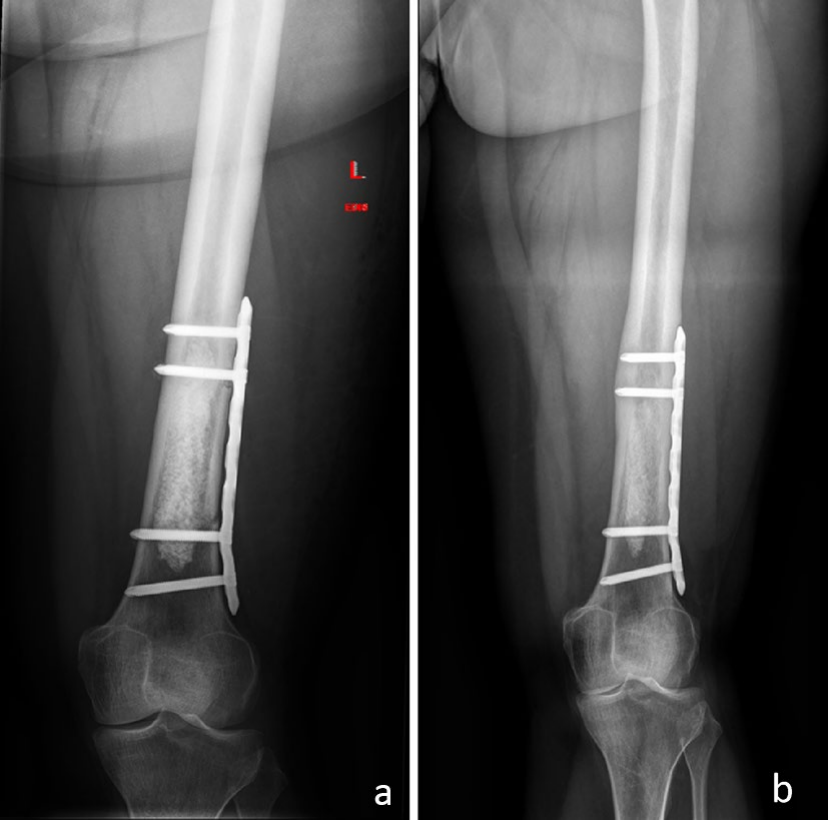
**a** X-ray of a 40-year-old female patient a few days after curettage of an enchondroma of the left distal femur with additional protective plate osteosynthesis. **b** Follow-up radiographs 12 months following index surgery showing integration but no resorption of the artificial bone graft substitute

After a mean follow-up period of 14.6 months, radiological consolidation of the cortex following curettage was observed in all patients after viewing standardized postoperative X-rays, which were performed 6 weeks, 3 months, 6 months, and 12 months after surgery. In case of doubt, consolidation was confirmed by an expert bone radiologist (J.I.). Cerasorb was totally resorbed in five patients (16.3%), altogether, as outlined in Fig. 3a and b. In the remaining patients (83.7%), partial resorption was observed, leaving the ABGS still clearly visible, as shown in Fig. 4a and b.

**Fig. 5:**
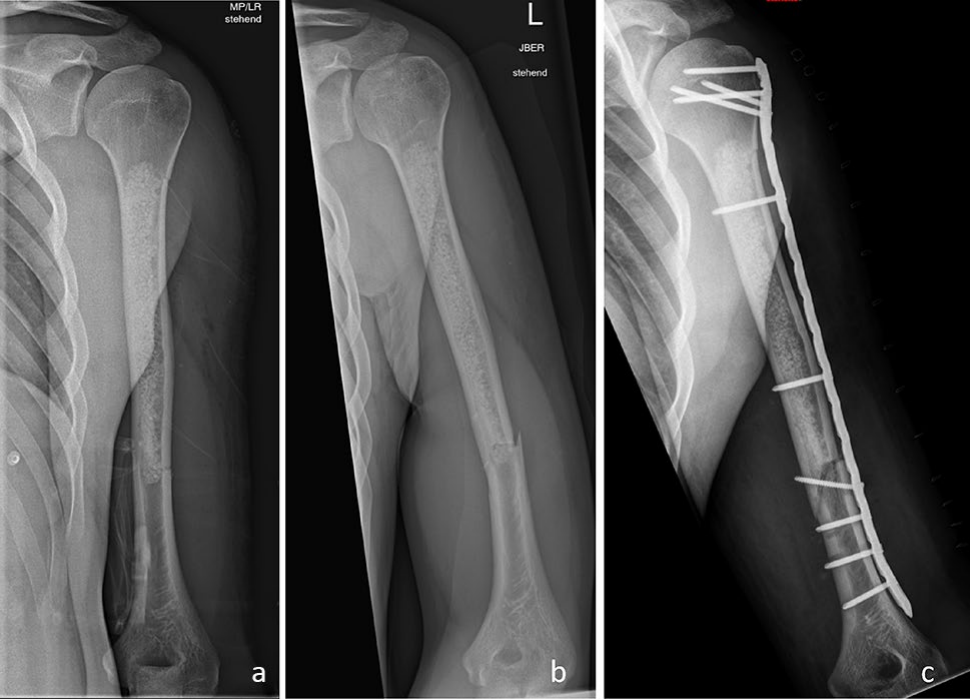
**a** X-ray of a 21-year-old male patient a few days after curettage of an enchondroma of the left humeral diaphysis, and **b** after a pathological fracture within the curetted area occurred after minor trauma. **c** Follow-up radiographs following plate osteosynthesis to stabilize the pathological fracture

In four patients, of whom two had a tumor in the distal femur and two in the humeral diaphysis, fractures occurred within 6 weeks after primary surgery, as outlined exemplarily in Fig. 5a and b. These lesions were rather large, with maximum diameters of 4.8 cm and 5.0 cm in the distal femur and 6 cm and 11 cm in the humeral diaphysis. In these cases, uncomplicated revision surgery with plate osteosynthesis was performed, which is shown exemplarily in Fig. 5c. No other complications were observed during the follow-up period. According to the classification system by Goslings and Gouma [17], the complications reported can be summarized as follows: none of the patients suffered grade 0, I, III, IV or V complications. Two patients presented with grade II complications, but showed total recovery after revision surgery. No local recurrences were observed during follow-up.

## Discussion

One of the most important findings of this study was that the ABGS Cerasorb is associated with adequate and fast bone consolidation and long durability. Moreover, the complication rate is rather small. Also, a minimal invasive, percutaneous approach would be possible, as performed frequently, for instance, in case of vertebroplasty or kyphoplasty. In theory, according to the instructions for use, the resilience of Cerasorb is supposed to allow for an unrestricted postoperative mobilization protocol. In the authors’ opinion, more restricted postoperative protocols have to be applied, depending on the individual case, defect size, and stability of the construct.

Complications associated with curettage and filling found in this study were due to a limited stability of the construct, leading to fractures when application was performed in a weightbearing bone, such as at the humeral diaphysis or the distal femur. In all cases, uncomplicated plate osteosynthesis was performed and healing was proper. However, little is known about stability and fractures after curettage and filling and thus, an important topic of future research will lie in the determination of stability properties of a tumor site, such as size and location that necessitate the use of protective plate osteosynthesis to avoid the occurrence of fractures. Hirn et al. found a strong correlation between size and volume of the tumor and postoperative fracture occurrence [20]. Kundu et al. proved that there was a correlation between size, volume, and localization of the tumor and the risk of postoperative fracture [21]. A retrospective case series performed by Perisano et al. in 2016 focused on indications for prophylactic osteosynthesis associated with the curettage of benign and low-grade malignant bone tumors in the distal femur [22]. They analyzed 12 patients treated by a single surgeon and recommended preventive osteosynthesis after curettage in case of very large lesions (> 5 cm, > 60 ccm) or high functional requirements, in obese patients and always, when local adjuvants such as ABGS were used. Moreover, studies showed that leaving the cavity unfilled involves an even higher risk of postoperative fracture and that the use of bone cement for filling yields the second highest fracture risk [20, 23, 24].

In 2017, Friesenbichler et al. performed a prospective, non-randomized study on 27 patients treated with the α-TCP Calcibon (Biomet, Warsaw, IN) in granular form for filling bony cavity defects after curettage of benign or low-grade malignant bone tumors with an average postoperative follow-up of 26 months. Radiological consolidation was observed in 23 patients after a mean follow-up of 6 months. Nonetheless, resorption was not seen by that time or even after 32 months of follow-up. Moreover, no local recurrences occurred [25]. The findings of this study were similar to those depicted in the present study, although with Cerasorb, complete resorption occurred in 16.3% of patients after a mean follow-up of 14.6 months. This is in line with current literature, as the ABGS Calcibon is similar to Cerasorb, but belongs to the group of α-TCPs, which are associated with a similar degree of solubility, but a slower biodegradation [14].

In 2006, Hirata et al. analyzed 53 patients with benign bone tumors who had undergone curettage and filling with β-TCP. In their patient collective, complete resorption rates of the β-TCP were higher than in the present study, even though the follow-up time was comparable [26].

There were several limitations associated with the present study. First, data collection was performed retrospectively. Second, the mean follow-up period was only 14.6 months, which is quite a short interval, so that long-term effects and time to full resorption in those patients where resorption was not complete after the end of follow-up are missing. This shows that to capture the full resorption profile of the ABGS Cerasorb, a longer follow-up interval is definitely needed in future research. Third, the patient collective was rather small and heterogenous, as a lot of different tumor entities localized in different bones were included. Moreover, mostly due to the high heterogeneity of data, the analysis was purely descriptive and statistical analysis could not be performed. Moreover, depending on the lesion's size, the costs for the artificial bone graft subtitutes used ranged from 100€ to 1000€.

In conclusion, based on our short-term observation, the β-TCP Cerasorb seems to be a reliable bone graft substitute with low complication rates and is a suitable alternative to autologous bone grafts or allografts. Nonetheless, it shows a tendency of delayed resorption.

## Data Availability

Data and materials are stored by the first author electronically.
